# Capgras Syndrome, Multiple Sclerosis, and COVID-19 Infection: A Case Report

**DOI:** 10.7759/cureus.53924

**Published:** 2024-02-09

**Authors:** Ayodele Atolagbe, Peterson Metellus, Stanley Nkemjika

**Affiliations:** 1 Department of Psychiatry and Behavioral Sciences, Kingsbrook Jewish Medical Center, Brooklyn, USA; 2 Department of Psychiatry and Behavioral Sciences, Interfaith Medical Center, Brooklyn, USA

**Keywords:** covid-19 induced psychosis, delusional disorder, covid 19, primary progressive multiple sclerosis, capgras syndrome

## Abstract

Capgras syndrome is a psychotic illness characterized by an unshaken false belief in having a close family member replaced by an imposter when there is no evidence of such. The patient described in this case report is a 68-year-old Caucasian female who presented with Capgras syndrome in the context of chronic progressive multiple sclerosis (MS) following an acute COVID-19 illness. She was treated with quetiapine with full resolution of symptoms.

## Introduction

Capgras syndrome is a type of psychotic disorder in which the patient holds a firm belief that a known, close individual (usually a loved one or family member) has been replaced by an identical imposter; it is also known as a delusion of doubles [1]. COVID-19 illnesses have become increasingly associated with psychiatric illnesses, including acute psychotic episodes and exacerbations of psychosis [2]. COVID-19 steroid and antibiotic treatment have also been associated with medication-induced psychotic disorders. Multiple sclerosis (MS) is a demyelinating neurologic disease often associated with psychiatric disorders such as major depressive disorder, anxiety, and psychosis [1,3]. We present a case of a 68-year-old female patient with known primary progressive multiple sclerosis who presented with first-episode psychosis characterized by Capgras delusions in the setting of COVID-19-positive nasal swab testing. Her symptoms developed acutely following inpatient intravenous steroid and antibiotic treatment for COVID-19 pneumonia. Her symptoms resolved completely after second-generation antipsychotic treatment.

## Case presentation

We present a case report of a 68-year-old Caucasian single mother of an adult daughter with a known diagnosis of primary progressive multiple sclerosis diagnosed in 2005 and stable on dimethyl fumarate. She had no other medical conditions and had no psychiatric or substance use history. She had developed an acute onset of paranoid delusions relating to her biological daughter’s identity after a 10-day acute inpatient hospitalization for COVID-19-related pneumonia, which was treated with a 72-hour course of intravenous methylprednisone, antibiotics, and remdesivir, followed by a five-day taper course of oral steroids and oral antibiotics. On her 10th day of inpatient stay, we transferred her to our facility for subacute rehabilitation.

Her daughter had urgently presented at the subacute rehabilitation facility on account of her mother’s hostile tone and aggressive speech during their telephone conversations. The patient insisted that the individual who had presented was not her daughter and refused to interact with or engage with her. All efforts to convince her to the contrary were unsuccessful. The daughter insisted that this was not her mother's baseline and described her mother as very loving and warm towards her. There was an episode of physical altercation between the patient and her daughter, who was also her next-of-kin, on the seventh day of her arrival at our facility, as observed by staff. A psychiatrist and a neurologist were consulted following this event.

A mental status examination revealed a well-groomed and cooperative elderly lady with fluent speech and a full and reactive attitude. Her thought process was mostly illogical and characterized by paranoid and persecutory delusional themes. She insisted that the lady who had presented was not her daughter but an identical imposter who wanted to steal her money. The patient perseverated with her real daughter being missing, insisted that unknown people had kidnapped her, and replaced her with this new person with the aim of stealing from her. She stated that unknown individuals had taken her daughter as punishment for her past misdeeds. Despite all the evidence shown to convince her otherwise, she persisted in endorsing these themes. There was no episodic confusion or fluctuating levels of consciousness in keeping with delirium. She had no reported changes in physical activity. There were no elevated mood and energy levels and no reduced need for sleep supportive of a manic episode, but she endorsed a significant inability to sleep during her acute inpatient stay and subacute rehabilitation.

**Figure 1:**
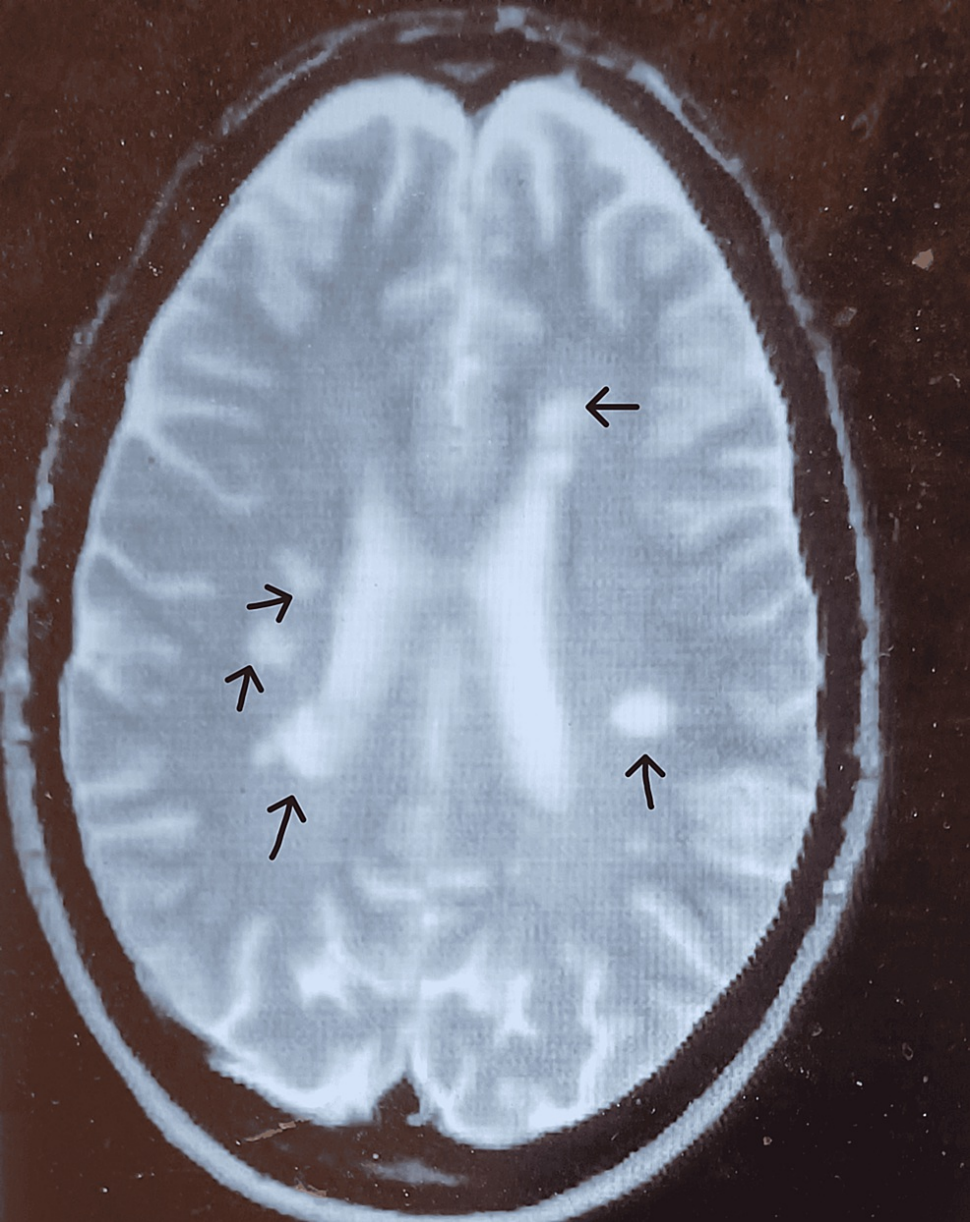
T2 MRI with arrows indicating the location of prior identified MS lesions. Arrows: the multiple MS lesions of the patient in the case report on MRI imaging. The lesions appear chronic, paraventricular, multiple, and bilateral - these characteristics have been linked with psychosis in MS.

The patient’s motor and sensory abilities remained at baseline, characterized by urinary incontinence and gait instability. She required an aide to assist her with chores and her ADLs. The patient had been COVID-19 positive at the onset of her admission and continued to test positive for COVID-19 by nasal swab until her 14th day of subacute rehabilitation. Her BMI was 18.1 and all vitals were normal. Chest X-ray imaging was negative for pneumonia. A brain MRI revealed multiple lesions in the bilateral and periventricular regions, all identical and chronic compared with her prior MRI imaging from a year ago (Figure 1). Her serum chemistry, liver, and renal function were normal. Urine toxicology was negative. Serum folate, vitamin B12, and iron panels were normal; VDRL testing for syphilis was negative; and cryptococcal and cytomegalovirus IgM and IgG titers were negative. Her Mini Mental Status Score was 24/30.

She commenced on Quetiapine XR 50 mg daily on her seventh day of subacute rehabilitation admission, increased to 100 mg daily on her 14th day of admission, and subsequently to 200 mg by the third week of admission, with reported improvement in sleep and a gradual resolution of delusional themes. She subsequently was noted to interact cordially with her daughter and resumed a familial relationship with her. She was discharged into her daughter’s custody at the end of her rehabilitation without any relapse noted at follow-up. She was maintained on Quetiapine XR 200mg long-term and remained psychiatrically stable at her outpatient clinic visits.

## Discussion

This report describes a patient with a multiple sclerosis diagnosis who presented with acute-onset delusions in keeping with Capgras syndrome following an acute COVID-19 illness treated with systemic steroids.

Multiple sclerosis is a common neurologic disorder characterized by demyelinating white matter lesions and varied motor and sensory presentations [1]. Psychiatric symptoms manifest in multiple sclerosis at a frequency two to three times greater than that of the normal population [1]. Psychiatric symptoms are being seen with increasing frequency in the setting of an acute COVID-19 infection [2]. Psychiatric symptomatology in multiple sclerosis also appears to be more female-preponderant and mood-related [1,3,4].

Capgras syndrome is a rare condition originally described by Capgras and Reboul-Lachaux in 1923 [5]. It has a prevalence of approximately 3% in hospitalized patients with psychosis [5], and most Capgras syndrome patients manifest first-episode psychosis, as seen in the patient presented [6]. Capgras syndrome is regarded as a psychiatric and neurological disease caused by structural and degenerative brain disease, as noted in strokes, dementia, and Parkinson's disease [3,4]. Two case reports have described a co-presentation of Capgras syndrome and multiple sclerosis [4], while none have described Capgras syndrome co-manifesting with COVID-19 and multiple sclerosis. No studies or case reports have shown an association between the trio of MS, Capgras syndrome, and acute COVID-19 infection.

MS has been more commonly linked with mood disorders such as major depressive disorder and anxiety [1,3]. Multiple sclerosis manifesting with psychotic episodes during its course is unusual. In patients with multiple sclerosis and psychiatric symptoms, lesions appear most commonly in the periventricular white matter of the right temporal and occipital lobes [7,8].

Corticosteroids and beta-interferon treatment for multiple sclerosis have been attributed to psychosis in multiple sclerosis [2]. However, the patient developed Capgras delusions following cessation of acute steroid and antibiotic treatment for acute COVID-19 infection, and her delusional symptoms persisted for weeks following acute hospitalization and discharge. Her delusions did not resolve until the commencement of antipsychotic treatment and a quetiapine dose increase [6].

A possible theory for Capgras syndrome in multiple sclerosis has been attributed to bifrontal and right hemispheric white matter lesions disconnecting the frontal lobe from the limbic system and temporal lobe regions, respectively [7,9,10]. The limbic system regulates emotions, while the temporal regions enable the recognition of familiar faces [7,10]. This leads to a resultant misidentification and loss of emotional recognition of previously known faces, as seen in Capgras syndrome [7-10]. An alternative explanation is a loss of connection between the visual cortex and the limbic system due to demyelinating lesions in the occipital and inferior parietal lobes as associated with prosopagnosia [7-10].

Multiple sclerosis patients manifesting with delusional symptomatology have been particularly associated with right hemispheric demyelinating lesions [2-5]. There are case reports of psychiatric symptoms with MRI findings showing no demyelinating lesions in the temporal lobe [8,9].

It appears that antipsychotic treatment is the mainstay of the management of multiple sclerosis and co-morbid psychosis [11]. Our choice of quetiapine was predicated on her reported sleep challenges and an underweight body mass index of 18.1, but successful treatment with aripiprazole has been reported [11].

## Conclusions

Patients with schizophrenia can have a concurrent diagnosis of multiple sclerosis, and vice versa. Patients with multiple sclerosis are more likely to develop schizophrenia and other psychiatric disorders. However, patients with primary psychotic syndromes such as schizophrenia are more likely to present with delusions and hallucinations at a younger age. They also tend to have a chronic course characterized by progressive functional impairment and psychosocial deterioration. Our patient's symptoms were presumably precipitated by an acute pulmonary infectious process (COVID-19 pneumonia); this makes a diagnosis of a psychotic disorder due to another medical condition per DSM-5 likely. Another consideration is medication-induced psychotic disorder due to recent exposure to intravenous/oral steroids, antibiotics, and antiviral treatment for COVID-19 pneumonia. Her prominent delusions without hallucinations and good baseline premorbid psychosocial functioning made a diagnosis of schizophrenia unlikely.

We suggest that older patients with multiple sclerosis presenting with first-episode psychotic symptoms undergo a full neurologic and psychiatric assessment. They also need a thorough medication review to rule out a medication-induced psychotic disorder. They need brain imaging to identify new brain lesions indicative of a multiple sclerosis flare. An active collaboration between neurologists and psychiatrists is essential in the treatment of multiple sclerosis patients manifesting with psychiatric symptomatology to improve their overall prognosis.
